# Exploring the role of auditory analysis in atypical compared to typical language development^☆^

**DOI:** 10.1016/j.heares.2013.09.015

**Published:** 2014-02

**Authors:** Manon Grube, Freya E. Cooper, Sukhbinder Kumar, Tom Kelly, Timothy D. Griffiths

**Affiliations:** Institute of Neuroscience, Medical School, Newcastle University, Framlington Place, Newcastle-upon-Tyne NE2 4HH, UK

**Keywords:** TD, Typically developing, DT, Dyslexic traits

## Abstract

The relationship between auditory processing and language skills has been debated for decades. Previous findings have been inconsistent, both in typically developing and impaired subjects, including those with dyslexia or specific language impairment. Whether correlations between auditory and language skills are consistent between different populations has hardly been addressed at all. The present work presents an exploratory approach of testing for patterns of correlations in a range of measures of auditory processing. In a recent study, we reported findings from a large cohort of eleven-year olds on a range of auditory measures and the data supported a specific role for the processing of short sequences in pitch and time in typical language development. Here we tested whether a group of individuals with dyslexic traits (DT group; *n* = 28) from the same year group would show the same pattern of correlations between auditory and language skills as the typically developing group (TD group; *n* = 173). Regarding the raw scores, the DT group showed a significantly poorer performance on the language but not the auditory measures, including measures of pitch, time and rhythm, and timbre (modulation). In terms of correlations, there was a tendency to decrease in correlations between short-sequence processing and language skills, contrasted by a significant increase in correlation for basic, single-sound processing, in particular in the domain of modulation. The data support the notion that the fundamental relationship between auditory and language skills might differ in atypical compared to typical language development, with the implication that merging data or drawing inference between populations might be problematic. Further examination of the relationship between both basic sound feature analysis and music-like sound analysis and language skills in impaired populations might allow the development of appropriate training strategies. These might include types of musical training to augment language skills via their common bases in sound sequence analysis.

*This article is part of a Special Issue entitled <Music: A window into the hearing brain>*.

## Highlights

• Auditory and language skills were tested in 28 11-year olds with dyslexic traits.
• Auditory processing of pitch, rhythm and modulation did not differ from controls.
• The pattern of correlation with language skills differed from that seen in controls.
• Differences in patterns of correlation merit further testing in prospective cohorts.

## 1 Introduction

A number of studies have sought links between auditory processing and language ability, both in typical and atypical language development. Dyslexia, a reading and spelling disorder that cannot be explained by low intelligence or lack of educational opportunity (Lyon et al., 2003) and Specific Language Impairment (SLI), a disorder of spoken language acquisition (Tomblin et al., 1997), have both been associated with deficits of auditory processing, but results have not been consistent in either case. The significance and specificity of the links between auditory processing and phonological, language and literacy skills (called language skills hereafter) remain to be better understood in both typical and atypical development.

To test the idea that auditory deficits lead to well-documented deficits in phonological representation in dyslexia (Snowling, 2000) that would then lead to reading and spelling impairments, a number of previous studies have sought deficits in basic auditory tasks using single sounds or pairs of sounds. Deficits in association with dyslexia or reading disability have been repeatedly reported for frequency discrimination in adults (e.g. Amitay et al., 2002; France et al., 2002; McAnally and Stein, 1996) and children (Halliday and Bishop, 2006a). Similarly, deficits have been shown for the perception of frequency modulation (FM) applied to pure-tone carrier stimuli at rates of 2 Hz and 40 Hz (adults: Ramus et al., 2003; Witton et al., 1998; children: Poelmans et al., 2011). These FM rates can be argued to be relevant to slow prosodic changes (over several hundreds of ms) and fast formant transitions (over tens of ms), respectively. Deficits in dyslexic children have also been shown for the processing of changes in amplitude, measured in the sensitivity for differences in rise time (Poelmans et al., 2011; Richardson et al., 2004). A number of studies demonstrated correlations in addition to a group difference, but typically across groups. In a re-analysis of previously published data on auditory deficits and correlations and reading abilities (Rosen, 2003) showed that these correlations would change or disappear when examined within as compared to across groups. Other studies failed to find group deficits, for instance in frequency or amplitude discrimination in dyslexic adults (Amitay et al., 2002; Hill et al., 1999), FM detection in children with dyslexia (Bishop et al., 1999) or at high risk of dyslexia (Boets et al., 2007), frequency discrimination in reading disability (Halliday and Bishop, 2006b), or backward-masking in adolescents or children with dyslexia or specific language impairment (Rosen and Manganari, 2001; Rosen et al., 2009), contrasting the above-mentioned reports of dyslexia and also SLI (e.g. Wright et al., 1997). The success of training and intervention strategies to improve language skills based on such one- or two-sound tasks remains a matter of debate (Gaab et al., 2007; but see Boyle et al., 2010; Fey et al., 2011; Given et al., 2008; Rouse and Krueger, 2004; Strong et al., 2011; Troia and Whitney, 2003), suggesting thatmay be the most relevant levels of auditory processing have not been tapped.

Studies going beyond basic single-sound perception showed dyslexia-related deficits in temporal-order judgements for pairs of tones (“low-high” or “high-low”) (Tallal, 1980) or other sounds (Ramus et al., 2003). Deficits in sound categorization based on more complex spectral changes in non-speech and speech sounds have been reported in children and adults with dyslexia (Vandermosten et al., 2011, 2010). A different set of studies have focused directly on the discrimination or identification of speech-type stimuli in quiet or in noise and demonstrated a significant relationship with both typical and impaired language development, including dyslexia and SLI (Watson and Watson, 1993, 2003; Ziegler et al., 2009, 2011, 2005). Studies assessing higher levels in generic, non-verbal auditory processing are rare to date, despite speech having a complex acoustic structure comprising of spectro-temporal patterns over multiple timescales, from the phoneme level (tens of milliseconds) to the sentence level (thousands of milliseconds) (Chi et al., 1999; Hickok and Poeppel, 2007; Jusczyk, 1999; Klatt, 1976; Liberman et al., 1956; Poeppel, 2003; Poeppel et al., 2004; Rosen et al., 2009; Schonwiesner and Zatorre, 2009; Scott, 1982).

In a recent study of a large, non-selected cohort of 210 typically developing individuals (age 11), Grube et al. (2012) tested the relevance of pitch and rhythmic sequence processing compared to more basic tasks of single-sound processing of pitch, time and modulation to phonological language and literacy skills. Their systematic approach based on multiple levels identified short-sequence analysis in pitch and time to be more strongly correlated with language skill than basic auditory processing, supporting the notion of the link between the two domains being in part a function of acoustic complexity (Rosen, 2003). Earlier speech work has demonstrated both pitch contour and rhythmic information to provide cues relevant to the parsing of the speech stream, in normal infants as well as in adults (Jusczyk et al., 1992; Smith et al., 1989). Recent work on basic pitch contour processing has reported deficits in dyslexic adults (Santurette et al., 2010), a deficit specific for the detection of local but not global changes in pitch contours in dyslexic children age 11 (Ziegler et al., 2012), as well as a specific, significant correlation for the more abstract “global” perceptual processing of transposed contours with reading ability in typically developing young adults (Foxton et al., 2003). Goswami and co-workers have looked at rhythmic amplitude modulation and musical rhythm processing in relationship to phonological language and literacy skills. The authors report group-level deficits in rhythmic amplitude modulation (rise time) and rhythmic change detection in sequences with varying degrees of musical meter in dyslexic compared to control children, in addition to significant correlations with phonological and literacy measures across groups (*n* = 64 in total; age 8–13). The authors further present regression analyses of metrical musical perception against basic auditory measures that could indicate group membership (Goswami et al., 2013, 2002; Huss et al., 2011; Overy et al., 2003; Richardson et al., 2004).

The present study tests for deficits and correlations with language skills for the same tasks of auditory processing used by Grube et al. (2012), from single-sound to sequence-based tasks, in a group of individuals with dyslexic traits (DT) compared to a group of typically developing (TD) individuals. The work tests the idea that there might be a difference in the relationship between auditory and language skills in addition to, or instead of an auditory deficit, as one possible underlying factor in atypical language development. We explore here the idea that atypical developers might be considered ‘different listeners’ rather than just ‘poor listeners’. Our a-priori hypothesis was that there may be a deficit in the yoking between auditory and language skills, which would predict a weaker relationship between aspects of sound perception and language skills than in typical development. Alternatively, the possible finding of stronger correlations would suggest a tighter coupling in language and auditory skills as a possible compensatory strategy for language-specific impairments. This is a first exploratory attempt, in a group of 28 individuals with dyslexic traits, who were part of the same whole-year group as the control group of 173 typically (TD) individuals. The TD group was drawn from the unimpaired group described in Grube et al. (2012). The auditory tasks ranged from single-sound to sound-sequence processing and assessed the domains of pitch, time and timbre. The language-based assessment of language skills used a set of six standardized tests of reading, spelling and related measures. Intellectual skills were also measured, as a potential confound and in order to identify individuals with dyslexic traits. The objective was, firstly, to test for the presence or absence of group differences in auditory skills and, secondly, to test for differences and commonalities in the links between auditory and language skills in the two groups.

## 2 Methods

### 2.1 Subjects

The present study sought differences in a group of individuals with dyslexic traits, the DT group (*n* = 28, 17 male; mean age = 11.46 years, SD = 0.26), compared to a typically developing (TD) group (*n* = 173, 67 males; mean age = 11.48 years, SD = 0.30). The DT group comprised individuals with dyslexic traits identified by a significant discrepancy between their full-scale IQ (FSIQ) and literacy-related scores, in accord with the DSM-IV (Diagnostic and Statistical Manual of Mental Disorders) discrepancy criterion for dyslexia. Language and intellectual skills were measured using standardized tests (described in Section 2.2) that transform the raw scores into age-independent standard scores with a normal distribution with a mean of 100 and standard deviation of 15. Sixteen individuals fulfilled the DSM-IV criterion of reading and spelling scores that were both lower than their FSIQ by 15 or more standard points; another 12 individuals had either a reading or a spelling score plus at least one associated standardized language measure (non-word reading, backward digit recall) with such a discrepancy of 15 or more standard points relative to their FSIQ. Both groups were part of a whole-year group (year 7; mean age 11.1 years, SD 0.3 years; *n* = 238; 99 male) at the comprehensive, non-selective St. Thomas More Catholic School, Gateshead, UK. The TD group consisted of 173 of the 210 individuals studied by Grube et al. (2012), excluding individuals with a full-scale IQ below 85 (1 SD from the mean; *n* = 34) or a verbal or non-verbal IQ below 70 (2 SD from the mean; *n* = 1), and those diagnosed with ASD/ADHD (*n* = 2) in order to provide a more comparable control group for the DT group. The research was approved by the ethics committee of Newcastle University.

### 2.2 Neuropsychological testing of language and intellectual skills

Tests of language and intellectual ability were administered one-to-one in a quiet room over a 1-hour period on a different day to the auditory sessions. As previously described in Grube et al. (2012), the six standardized tests of phonological language and literacy skills (here referred to as language tasks) were: 1) written rhyme decision (the child reads a list of pairs of words and decides silently for each one whether they rhyme or not: Psycholinguistics Assessment of Language Processing in Aphasia, PALPA (Kay et al., 1992)); 2) spelling (the child writes down the spelling of spoken words: Wechsler Individual Achievement Test, WIAT-II^uk^ (Wechsler, 2005)); 3) word reading (the child reads aloud a list of written words: WIAT-II^uk^); 4) non-word reading (the child reads aloud a list of nonsense written words: WIAT-II^uk^ – “pseudoword decoding”); 5) non-word repetition (the child repeats back spoken nonsense words: Working Memory Test Battery for Children, WMTB-C – “nonword list recall” (Gathercole and Pickering, 2001)); 6) backward digit recall (the child reproduces in reverse order sequences of digits: WMT-C). Full-scale IQ (FSIQ) was assessed by the Wechsler Abbreviated Scale of Intelligence (WASI (Wechsler, 2005)), which includes 2 verbal and 2 non-verbal subtests. Verbal IQ is assessed by the vocabulary subtest (the child orally defines spoken words) and the similarities subtest (the child orally describes the similar concept that binds together two spoken words). Non-verbal IQ is assessed by the block design subtest (the child produces a copy of a 2D pattern with coloured blocks) and the matrix reasoning subtest (the child indicates a picture from a selection that will complete the pattern presented).

### 2.3 Auditory testing

**Fig. 1:**
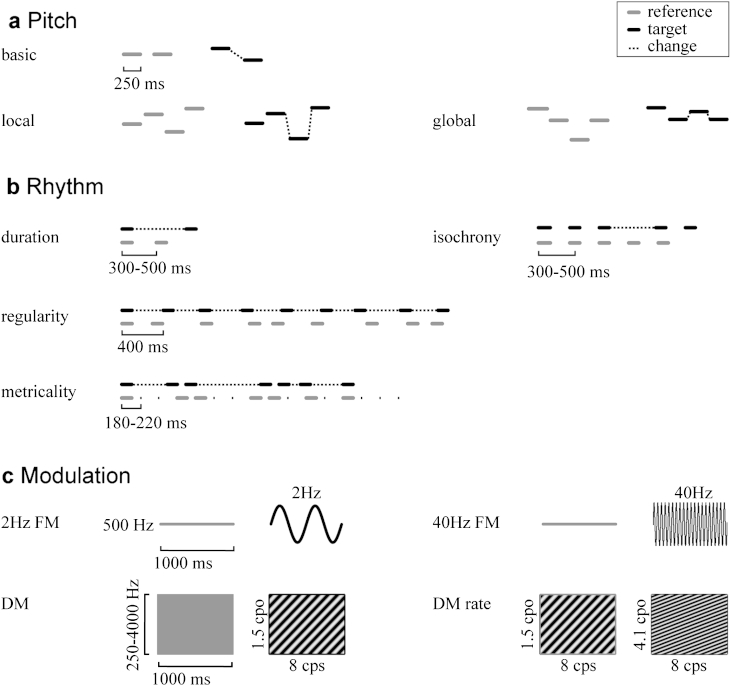
Schematic depiction of auditory tasks as in Grube et al. (2012). For each task, one reference and one target example are illustrated with their relevant features; abscissa and ordinate depict time and frequency, respectively (throughout but with varying scales). a Pitch: basic change detection (pairs of tones); local and global pitch change detection (short sequences); key violation: not shown. b Rhythm: single time-interval duration discrimination (pairs of tones); isochrony-deviation detection (short sequences); regularity detection and metrical pattern discrimination (longer sequences). c Modulation: 2 Hz and 40 Hz frequency modulation (FM) detection; dynamic spectral modulation detection (DM) and rate discrimination (DM rate) (dark stripes representing peaks moving across frequency and time). Abbreviations: cpo, cycles per octave; cps, cycles per second. Note that the basic change detection in pitch, the duration discrimination, and the FM and DM detection and discrimination tasks would be classified as basic, single-sound based tasks, as opposed to the remaining tasks testing aspects of sequence analysis in pitch and time.

Auditory testing was performed in a quiet classroom environment, one class at a time (*n* = 16–30). The class was instructed by the lead researcher for one task at a time; task understanding and compliance were assured by group-level instructions, practice trials, and questions addressed to the whole class, for which each individual was required to raise their hand according to what they perceived to ensure as best as possible that the children understood the task. Each pupil then performed the task independently on their own, running Matlab^®^-based standalone executables on individual setups (computer, external soundcard, closed headphones). Four pitch perception tasks, 4 rhythm and timing tasks, and 4 tests of timbre perception based on modulation (Fig. 1) were performed in three sessions of 60–75 min each. All tasks used a two-alternative forced-choice paradigm. Most tasks used a 2-down-1-up adaptive tracking algorithm estimating the 70.9% correct threshold (Levitt, 1971), except the three pitch sequence tasks. Those had fixed difficulty levels with the number of correct responses being the most immediate outcome measure and used in the present analysis; for further task details beyond the descriptions below see (Grube et al., 2012).

#### 2.3.1 Pitch (Fig. 1a)

The first three pitch tasks used 250-ms pure-tones, the fourth used synthetic-piano melodies. The *basic pitch change detection task* required the subject to indicate which of two pairs of pure-tones included a change in frequency. The *local and global change detection tasks* (40 trials each, same-different) required the subject to indicate whether two four-tone sequences were “the same or different” (adapted from Foxton et al., 2003). In the local task, the change in frequency of one note preserved the patterns of “ups and downs”, but not in the global version the change in note caused also a change in melodic pattern. The *key violation detection task* from the Montreal Battery for the Evaluation of Amusia (Peretz et al., 2003) required the subjects to indicate whether two melodies were “the same or different”, with the change in one note violating the key structure. The first three tasks test the perception of pitch changes found in either speech or music, whilst the fourth is specific to the tonal structure of Western music.

#### 2.3.2 Rhythm (Fig. 1b)

All four rhythm and timing tasks (Grube et al., 2010) used 500-Hz 100-ms pure-tones. The *basic, single-interval task* required subjects to indicate which of two tone pairs comprised the “longer gap”. In the *isochrony-deviation detection task*, subjects were required to indicate which of two otherwise isochronous five-tone sequences contained a lengthening or “extra gap”. In the *regularity detection task*, subjects were required to indicate which of two nine-tone sequences was “overall more regular”. The reference had an average irregularity of ±30%, due to shortening or lengthening of individual intervals by 15–45% each, rendering the beat imperceptible (Madison and Merker, 2002). The target had 0% irregularity initially, which increased adaptively. In the *metrical pattern discrimination task,* subjects were required to decide which of three rhythmic sequences was “different, or wrong” due a distortion in the rhythm. The reference had a metrical beat of 4 induced purely by the temporal spacing of 7 tones, with phenomenally accented tones occurring on each of the 4 intended down-beat locations, following Povel and Essens (1985)'s behavioural observations model of metrical beat strength. To minimize stimulus uncertainty, an extra reference was presented first. The target (third or second) had a change in timing such that the long intervals were no multiples of the underlying beat: the pattern would sound “wrong”. Two intervals were shortened and two lengthened (by the same percentage and thus cancelling out in total sequence length), with the four available combinations applied in rotating manner (for more details see Grube and Griffiths, 2009). Across tasks, inter-onset-intervals ranged from 180 to 660 ms, corresponding to time intervals between stress events in speech (Grabe and Low, 2002; Rosen, 1992; Scott, 1982) and musical beats (Drake et al., 2000; London, 2004).

#### 2.3.3 Timbre (Fig. 1c)

The four tasks of timbre perception included two FM detection tasks, implicated in reading ability previously (Talcott et al., 2000; Witton et al., 1998), plus dynamic-modulation (DM) detection and discrimination tasks based on spectral-temporal modulations relevant to speech (Chi et al., 1999; Schonwiesner and Zatorre, 2009). In the *FM detection tasks*, subjects were required to identify a tone modulated at a rate of 2 Hz, sounding “ringing or wobbly” or 40 Hz, sounding “rough” against a “flat-sounding” unmodulated 500-Hz reference. Tone duration was 1000 ms including 20-ms gating times. The threshold was measured in modulation index [MI, defined as the ratio of maximum frequency deviation (Hz) to modulation frequency (Hz)] was 3.5 for the 2 Hz FM (equalling ±7 Hz maximum frequency deviation for the carrier) and 0.16 for the 40 Hz FM (equalling ±6.4 Hz maximum deviation).

In the *DM detection task*, subjects discriminated a modulated (“alien or laser-like”) target sound against an unmodulated reference. Sounds were composed of 100 logarithmically spaced components per octave, over a range from 250 to 4 kHz, whose amplitudes were sinusoidally modulated dynamically in frequency (spectral) and time (temporal) with a rate of 1.5 cycles per octave (cpo) and 8 Hz, respectively. In the *DM discrimination task*, subjects discriminated a target sound with a higher spectral modulation density (in cpo) against a reference with a spectral modulation rate of 1.5 cpo, and a temporal modulation rate of 8 Hz, at a modulation depth of 0.75. Stimulus duration was 1000 ms and amplitude peaks were moving up in frequency over time.

### 2.4 Statistical data analysis

The data from the DT group were analysed in comparison to those from the TD group. A small number of data points were missing due to occasional absence or failure to complete a test. Within the DT group, one out of the 28 participants had no spelling score; one had no rhyme decision, non-word repetition or backward digit recall score; one missed three out of four rhythm tasks; three missed one or more of the modulation tasks. Within the TD group, there was an average of 7% missing per measure. Each analysis was performed on all of the available data.

Firstly, we tested for group differences in auditory, language, and intellectual ability scores. Significant between-group differences were determined using the Mann–Whitney *U*-Test, with Bonferroni correction applied for the testing for differences in multiple measures in parallel (auditory, 12; language, 6; intellectual, 3; total, 21).

Secondly, we tested for correlations between auditory and language measures and group differences in correlations. Correlations were estimated with Spearman's rho, as a number of measures showed a significant deviation from a normal distribution using the Lilliefors version of the Kolmogorov–Smirnoff Test for composite normality (for descriptive statistics see Tables 1 and 2). Correlations were in all cases corrected for effects of non-verbal IQ, which was partialled out. As in Grube et al. (2012), a one-tailed Spearman's rho was used as a general, positive correlation between auditory and language skills was predicted. Bonferroni correction was applied to avoid false-positives for the testing of multiple measures in parallel. Differences in the correlations in the DT group compared to the TD group were tested statistically using bootstrapping analysis using 1000 iterations. On each iteration, the Spearman's rho correlation coefficient between the two variables of interest was obtained from a randomly chosen TD subsample the same size as the DT group. The difference in correlation coefficient between the DT and the TD group was tested for significance at the level of *p* ≤ 0.05 (two-tailed) by z-score evaluation of the DT group's rho value compared to the 1000 rho values obtained from the bootstrapping carried out for the TD group: *z* ≥ 1.96 and *z* ≤ −1.96, for significantly higher or lower, respectively. The same *z*-score based evaluation was carried out for the mean correlation coefficient across language tasks, in order to obtain one overall measure of correlation for each of the auditory tasks. The evaluation of correlation coefficients was performed only after partialling out non-verbal IQ in order to avoid any effect of the group difference in IQ.

## 3 Results

### 3.1 Auditory, language and literacy, and intellectual ability scores

**Fig. 2:**
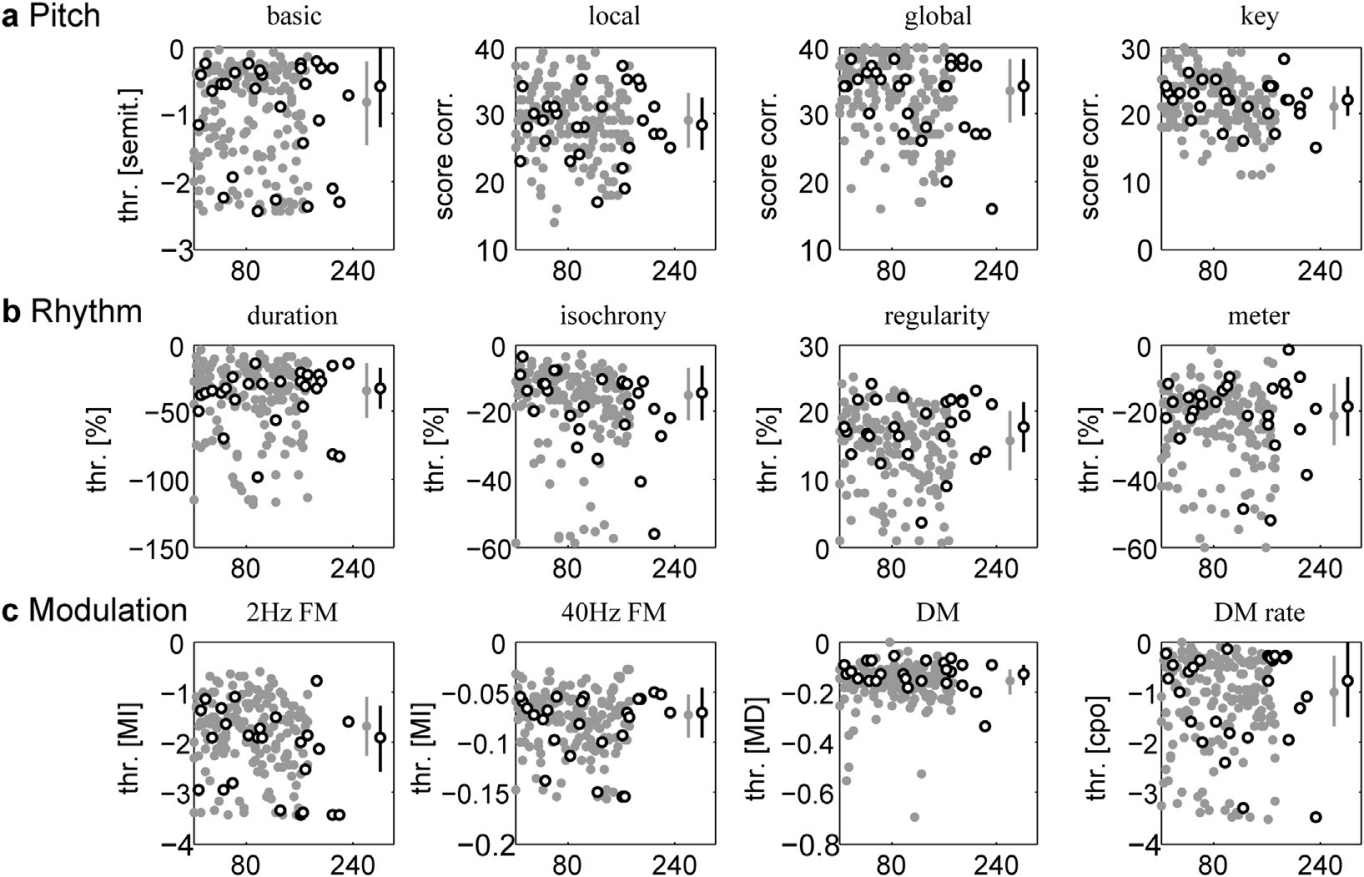
Raw auditory data for the group of individuals with dyslexic traits (black open circles) compared to the larger control group of typically developing individuals (grey filled circles). a Pitch; b Rhythm; c Modulation. Individual scores are plotted in the order of ability banding along the abscissa, using the same subject index of 1–238 as Grube et al., 2012. Group medians and mean absolute deviations (see Table 1) are shown by dots with error bars at the far right within each subplot. Note that for all of the measures for which lower values (thresholds) indicate better performance, i.e. all measures expect for the three pitch tasks using score correct and the regularity detection task, signs were reversed so that in all plots “higher up” means “better”. Abbreviations: thr., threshold.

Amongst the twelve auditory measures of pitch, time and rhythm and timbre perception, no significant deficit was found in the DT group compared to the TD group (Table 1; Fig. 2). The one task on which the DT group performed borderline significantly poorer than the TD group was 2-Hz FM detection (*p* = 0.056 before Bonferroni correction, *U* = 1917, *z* = 1.91; Mann–Whitney *U* Test). The only significant differences observed between groups were in fact two comparisons with effects in the other direction reflecting better performance: regularity as well as DM detection thresholds were both lower in the DT compared to the TD group (both *p* < 0.01, not surviving Bonferroni correction; *U* = 3217 and 3140, *z* = 2.61 and 3.14; Mann–Whitney *U*-Test). A few other measures showed a trend in the same direction of better performance in the DT compared to the TD group, but did not approach significance. There is the possibility of missing a true effect due to the small sample size of the DT group; in order to find a significant effect this group would ideally be of similar size (>130) to the TD group. Moreover, we use Bonferroni correction for multiple comparisons. However, the trend toward better performances in the DT compared to the TD group, with the exception of 2-Hz FM detection suggests that a group-level deficit in auditory processing is unlikely (Fig. 2).

**Fig. 3:**
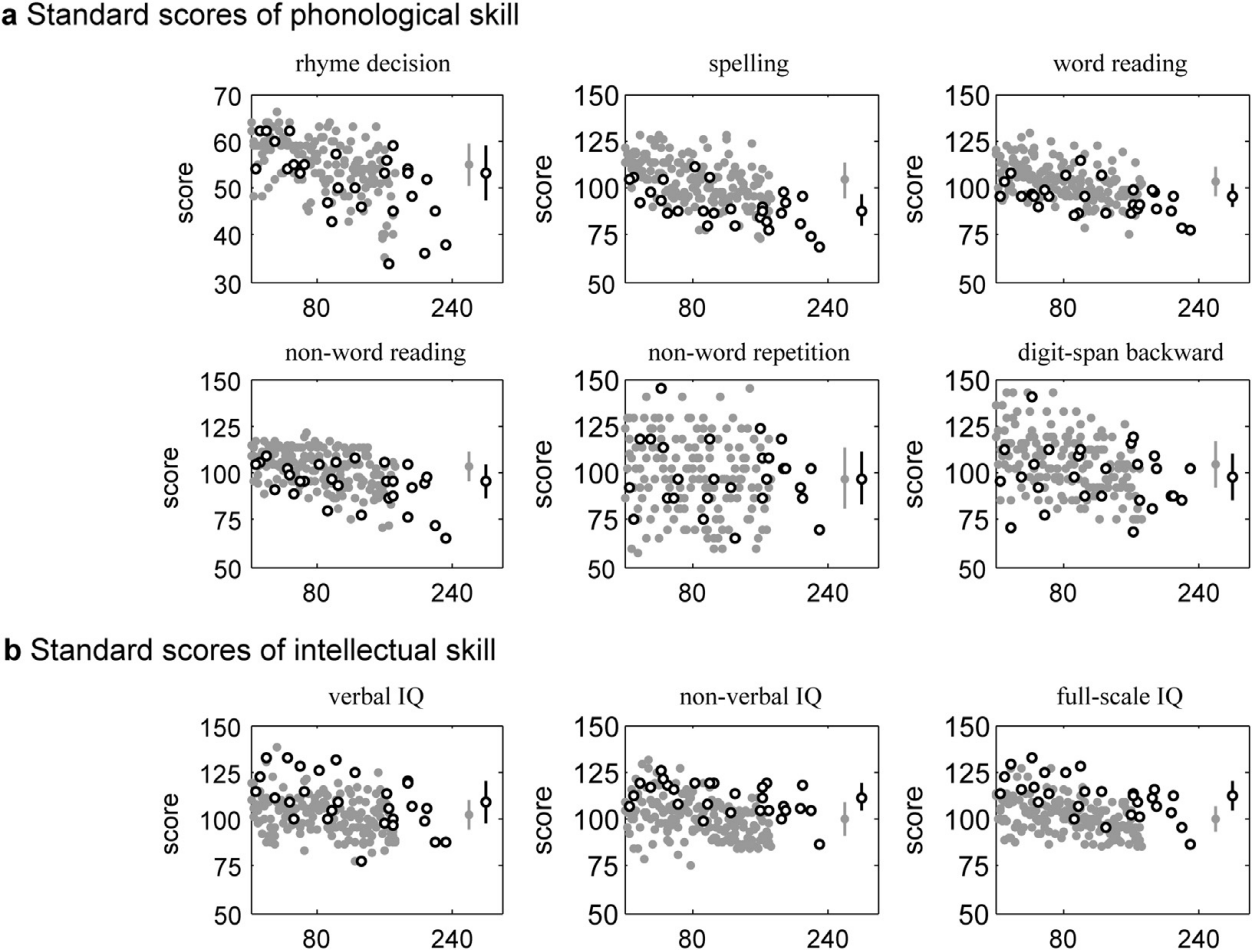
Language and literacy scores and intelligence measures for those with dyslexic traits (black open circles) compared to the larger group of typically developing individuals (grey filled circles). There was a highly significant difference between groups for the reading (wrd), spelling (spl) and non-word reading (nrd) scores, as well as for the full-scale IQ (FSIQ), non-verbal (or, performance) IQ (PIQ), and verbal IQ (vIQ) but in the other direction (*p* < 0.001 before and after Bonferroni correction for multiple comparisons; Mann–Whitney *U* Test). Plot details as in Fig. 2.

Amongst the language measures, a highly significant difference between the TD and DT groups was found for spelling, reading and non-word reading, with lower scores being achieved by the DT group (all three with *p* < 0.001 and surviving Bonferroni-correction for multiple comparisons; *U* = 1229, 1626 and 1799; *z* = −5.21, −4.16, and −3.58; Mann–Whitney *U*-Test; Table 2; Fig. 3). In addition, there was a significant effect of lower scores in the DT compared to the TD group for the rhyme and backward digit recall tasks (*p* < 0.05, not surviving Bonferroni correction; *U* = 1874 and 2021; *z* = −2.32 and −2.30; Mann–Whitney *U*-Test). The one task showing not even the slightest trend for poorer performance in the DT compared to the TD group was that of non-word repetition, which is the task relying most crucially on auditory information.

A highly significant difference between TD and DT groups was further found for the verbal, non-verbal, and full-scale IQ, where higher scores were achieved by the DT than the TD group (all three, *p* < 0.001 and surviving Bonferroni-correction; *U* = 3696, 4158 and 4158; *z* = −3.04, 4.66 and 4.66; Mann–Whitney *U*-Test; Fig. 3). In order to test whether the absence of an auditory deficit in the DT group might be related to the difference in IQ, a between-group comparison was performed for a closely matched subsample of TD individuals (matched in gender as well as FSIQ mean and variance), and in addition by testing for correlation between the auditory measures and IQ. No significant effects of IQ were found.

### 3.2 Correlations between auditory and language skills

The main objective of this study was to seek deviations in the pattern of correlations between auditory and language skills in the DT group compared to the TD group, which tests the hypothesis that dyslexia may not simply be a function of auditory impairment but associated with differences in the relationship between auditory and language skills. Correlations were analysed between the task-specific measures of auditory and language skills (Tables 3 and 4), and evaluated in comparison to those observed in the TD group after partialling out non-verbal intelligence (Tables 4 and 5).

The correlations observed in the TD group were very similar to those reported by Grube et al. for the more inclusive group (2012), i.e. very little affected by the application of a lower IQ limit to match the DT group and excluding three subjects with ASD/ADHD in the present analysis. We mention in the text those correlations within the DT group that had a rho ≥0.22 (i.e. explaining at least 5% of the variance) after partialling out non-verbal intelligence, and were significant at the level of *p* < 0.05 before Bonferroni correction, following the same criteria as in our previous report (Grube et al., 2012). Whilst Bonferroni correction is the most conservative method of avoiding “false positives” due to multiple comparisons, the exploratory nature of the present study and the comparison of a relatively small sample to a relatively large one support an inclusive presentation over an overly strict exclusive one which may overlook potential true correlations due to lack of power. We tested for significant differences in correlations in the DT vs. TD group by bootstrapping of TD subsamples for those correlations that fulfilled the criteria (*p* ≤ 0.05 and rho ≥0.22) in at least one of the groups, and for the mean correlation coefficients across language measures for those auditory measures that showed at least one such significant individual correlation. Performance on the two pitch tasks was strongly correlated in both groups (TD: rho, 0.69, *p* < 0.001, *n* = 164; DT: rho, 0.49, *p* < 0.01, *n* = 28) and the correlations with language skills might be due to a common mechanism of pitch sequence processing. To assess such a mechanism, we used principle component analysis to extract the first component as a combined score, which explained 84% and 76% of the variance in the TD and the DT group, respectively, and analysed also the correlations for this combined measure.

Whilst significant correlations between auditory and language measures in the TD group were predominantly found for the three tasks of short-sequence analysis in pitch and time (Table 5; see also Grube et al., 2012), the DT group showed a somewhat different pattern. A tendentious relative decrease in correlations was seen for the measures of short-sequence processing, i.e. the local and global pitch sequences and the isochrony tasks. The largest, near-significant decrease in correlation compared to the TD group (according to bootstrapping analyses on 1000 subsamples matched in size to the DT group) was that for the correlation between the local change-in-pitch sequence tasks and non-word reading (*z*, −1.91). Conversely and more strikingly, there were a number of significant, moderate correlations with rho values >0.3 in the DT group that were either lower or absent in TD group. Specifically, those were found for the auditory single-sound tasks of basic pitch-change, 2-Hz FM detection and DM discrimination. The largest, near-significant increase in correlation compared to the TD group (according to bootstrapping analyses on 1000 subsamples matched in size to the DT group) was that for the correlation between 2 Hz FM detection and word-reading (*z*, 1.91). For both the 2-Hz FM detection and the DM discrimination task, there was an overall increase with the language measures in the DT compared to the TD group, reflected in a significant difference in the mean correlation coefficient (*z* = 2.35 and 2.41), a measure of the overall relevance of each auditory task to language skills.

### 3.3 Results summary

The DT group showed no significant impairment in auditory processing scores compared to the TD group in any of the measures of pitch, rhythm or modulation processing; however, they had significantly lower dynamic modulation thresholds. For the phonological language and literacy measures, the DT group performed significantly poorer than the TD group on reading, spelling and non-word reading (as could in part be expected by the use of these measures for identification). They scored significantly higher on the estimates for FISQ, verbal and non-verbal IQ than the TD group. The difference in IQ did not explain the absence of auditory deficits.

Correlations between auditory and language skills were of similar magnitude in the DT group and TD group, with Spearman's rho correlation coefficients up to 0.4, but showing a somewhat different pattern. The DT group exhibited a relative increase in correlations for some of the basic, single-sound tasks, most strongly so for FM-2 Hz detection and DM discrimination, compared to the TD group, and a relative lack in significant correlations for the sequence tasks, though this may in part be due to a lack of statistical power related to sample size.

## 4 Discussion

The present study explores the idea that correlations between auditory and language skills may in part be the same but in part differ in typical compared to atypical development. We tested here a range of auditory and language skills in a group of individuals with dyslexic traits for differences in comparison to a control group of typically developing individuals, and for commonalities and differences in the pattern of correlations between auditory and language skills. We assessed auditory and language skills in 28 eleven-year olds with dyslexic traits, the DT group, in comparison to 173 typically developing subjects, the TD group, who underwent the same systematic assessment (Grube et al., 2012). The auditory assessment included tasks of pitch, time and rhythm, and timbre (modulation) processing, using acoustic stimuli that ranged from basic, single sounds to sound sequences. The assessment of language skills used a combination of six standard tests of phonological language and literacy abilities. Firstly, there was no group-level deficit in the auditory tasks in the DT compared to the TD group that could explain their language difficulties. Secondly, the existence and specificity of the links between auditory and language skills was compared between the two groups. The correlations found in the DT group were of similar, small-to-moderate effect size as in the TD group, with rho values up to about 0.4, but showed an in part different pattern.

### 4.1 Language, auditory and intellectual skills

The DT group comprised a sample of just below 12% of the whole year-group, consistent with the reported frequency of occurrence of developmental dyslexia (Lewis et al., 1994; Meltzer et al., 2000). Highly significant group differences between the DT group and the TD group were observed for the measures of reading, spelling and non-word reading, and borderline significant ones for rhyme decision and backward digit recall. The DT group further exhibited significantly higher scores of intellectual skills, both non-verbal and verbal, as well as a significantly higher composite full-scale IQ. The use of a within-subject discrepancy criterion may explain the difference in IQ. It remains remarkable that, despite the overall higher IQ in the DT group, three of the language measures were significantly impaired in comparison to the TD group. However, no significant deficits in auditory skills were found in the DT group compared to the TD group in the three domains of pitch, time and rhythm, and modulation processing, except for a marginally significant trend for the slow (2-Hz) frequency modulation task. To the contrary, a number of auditory measures showed a tendency toward better performance in the DT compared to the TD group. This effect and the absence of group-level deficits could not be explained by the group difference in intellectual skills, as there were also no group deficits compared to an FSIQ-matched TD subsample and no significant correlation between the auditory measures and intelligence in either the DT or the TD group.

The dyslexic traits seen in the present DT group comprising 28 out of a cohort of 238 individuals in total thus cannot be simply attributed to a fundamental auditory deficit. With the only exception of a marginally poorer performance for the 2-Hz FM detection task, no group deficit was found in basic pitch or time-interval discrimination, the detection of a simple frequency modulation, or dynamic spectral modulation detection or discrimination. Some previous reports have demonstrated such deficits for frequency discrimination (Amitay et al., 2002; France et al., 2002; Halliday and Bishop, 2006a; McAnally and Stein, 1996), FM detection (Poelmans et al., 2011; Ramus et al., 2003; Vandermosten et al., 2011; Witton et al., 1998) or related spectral processing tasks using speech and non-speech stimuli (Vandermosten et al., 2011, 2010), but others have not (Amitay et al., 2002; Bishop et al., 1999; Boets et al., 2007; Halliday and Bishop, 2006b; Hill et al., 1999; Rosen and Manganari, 2001). For the DM detection task in the present study in fact, there was a trend toward better performance in the DT compared to the TD group, consistent with an increase sensitivity to spectral discrimination in dyslexic children reported by Serniclaes et al. (2001). There were also no significant deficits in sequence processing in either pitch or time, which might have been expected based on studies suggesting a relevance of sound sequence or suprasegmental analysis to reading (e.g. Grube et al., 2012; Huss et al., 2011; Ziegler et al., 2012). Whilst the criteria used to identify the present sample of subjects with dyslexic traits was in accord with DSM-IV, this group does not suffer from either the auditory or a phonological deficit that has been demonstrated in previous studies. The current data thus do not directly support the hypothesis that auditory deficits cause a lack of phonological awareness and reading difficulties in this population.

### 4.2 The link between auditory and language skills

The role of auditory processing in language development has been controversial (Rosen, 2003), and we suspected that rather than being attributable to a deficit in auditory processing, dyslexic traits may, in part, be associated with a difference in the link between auditory and language skills. Previous work on correlations between the two domains tended to focus on one specific aspects of auditory processing, and some supported a link with typical or atypical language development (e.g. Foxton et al., 2003; Goswami et al., 2002; Huss et al., 2011; Richardson et al., 2004; Talcott et al., 2000; Tallal, 1980; Temple et al., 2003; Witton et al., 1998; Wright et al., 1997), whilst others did not (e.g. Boets et al., 2007; Rosen et al., 2009). A systematic investigation that compares typical and atypical development across a range of tasks and different levels of complexity has not been performed before. The present data support the existence of a limited relationship between auditory processing and language skills in the two groups tested here, with an in part different pattern in correlations in the group of individuals with dyslexic traits compared to the typical developers.

### 4.3 The role for basic auditory processing

In the present group of TD individuals, drawn from the same population as studied by Grube et al. (2012), correlations between single-sound tasks and language skills were not only very low in comparison to previous reports (c.f. Corriveau et al., 2010; Poelmans et al., 2011; Ramus et al., 2003; Talcott et al., 2000; Witton et al., 1998) but practically absent. There was no single correlation between language measures and the basic pitch or duration or any of the single-sound modulation tasks that was significant and explained more than 5% of the variance. In the DT group however, there were a number of correlations between the basic, single-sound tasks and language measures, in particular for the 2-Hz FM detection and the DM discrimination task, and both of those showed a significant increase in the mean correlation coefficient. FM detection at a modulation rate of 2-Hz can be argued to be relevant to suprasegmental processing, whilst the detection of moving spectral peaks at is relevant to the analysis of spectral features like formants. 2-Hz FM detection has been shown before to correlate with non-word reading in a group of typically developing 10-year olds (Talcott et al., 2000) as well as in typically developing and dyslexic adults (Witton et al., 1998). The lack of correlations across both FM and both DM, as well as the basic pitch and duration tasks, found in the present TD group suggests that, as discussed by Grube et al. (2012), despite the presence of the corresponding features in speech (Chi et al., 1999; Jusczyk, 1999; Klatt, 1976; Liberman et al., 1956; Rosen et al., 2009; Schonwiesner and Zatorre, 2009; Scott, 1982), highly accurate auditory analysis of these features might not be needed to process the corresponding cues adequately in typical development. In the DT group, however, significant, moderate correlations were observed for both the 2-Hz FM detection and the DM discrimination, supporting a tighter coupling between sound processing and language skill than in typical development.

### 4.4 The role for auditory sequence analysis

Of the tested levels of auditory processing, short-sequence analysis in pitch and time were demonstrated to be most relevant to language skills in the TD group studied here, in accord with our previous report (Grube et al., 2012). There were moderate correlations between the language skills and the local and global change-in-pitch tasks using short melodies, as well as the detection of a deviation from a short, otherwise isochronous rhythm. The underlying processes of auditory-sequence analysis can be thought to be relevant to the ‘parsing’ of the speech stream in real-time (Jusczyk, 1999), consistent with the perceptual organization of phonological representations starting at the higher, suprasegmental level before the analysis of phonemes (Goswami et al., 2002; Metsala and Walley, 1998). In the DT group of the present study, the corresponding correlations between measures of language skills and the processing of short sequences were less prominent and hardly significant, though not absent and the lack of significance may be related to sample size. This supports a universal relevance for sound-sequence analysis in speech and language skills. An important study by Kraus and colleagues demonstrated a relationship between reading ability and accuracy of auditory processing of the speech stream, with specific focus on the amplitude envelope and measured in electrophysiological brain-stem responses (Abrams et al., 2009). Speech processing has subsequently been linked to oscillatory processes in the brain at relevant periodicities at the prosodic and the syllable level (Ghitza, 2013; Giraud and Poeppel, 2012). Further, Lehongre and coworkers (Lehongre et al., 2013, 2011) have demonstrated abnormalities in the oscillations in dyslexia. Recent work by Leong and Goswami (2014), appearing in this special issue, tested rhythmic entrainment at the timescales for prosody, syllables, and phonemes in dyslexic adults and controls using metrically regular nursery rhymes. Whilst the dyslexics exhibited a different phase angle than controls at the syllable level (5 Hz), phase-locking to the amplitude fluctuations was equally strong in both groups (Leong and Goswami, 2014).

Grube et al. (2012) suggest that the correlations between auditory sequence processing and language in the larger group of TD subjects are consistent with such mechanisms providing a common basis for music and speech (Goswami, 2010; Overy et al., 2003; Patel et al., 2005). The link may be tighter in the domain of rhythm than pitch (Grube et al., 2013, 2012; Hausen et al., 2013).

### 4.5 Auditory skills as markers of dyslexia?

The DT group had lower word reading, spelling and non-word reading scores than predicted by their own intellectual ability scores, but also in absolute terms when compared to the TD group. If auditory deficits were the determining causal factor for dyslexia, this would predict low auditory performance compared with the typically developing sample. This has been demonstrated in previously studied samples. Here the raw scores showed no significant impairment in any of the auditory measures in the DT group. This absence of group-level deficits is in contrast to some previous reports but not others, supporting the notion that an auditory processing deficit is not necessary (nor sufficient) to cause dyslexia or SLI (Rosen, 2003; Thomson et al., 2006; Bishop et al. 1999).

Despite the absence of group deficits, we have demonstrated differences in the task-specific correlations in the DT group compared to the TD group. In contrast to our a priori suggestion, there was a relative *increase* in correlation, for the basic tasks of slow (2-Hz) FM detection and DM discrimination with language measures. Such an increase would, rather than a lack of “yoking” between auditory and language skill development, suggest a somewhat tighter coupling than in typical development. This might reflect a compensatory use of auditory skills to overcome difficulties with reading and spelling. For the tasks of short sequence analysis in contrast, there was a relative lack of significant correlation with language skills, which may reflect less relevance than in typical development at this age. This may, however, be related to statistical power.

The findings are specific to the present group of 11-year olds with dyslexic traits and can by no means be generalized to auditory processing in dyslexia in its entirety, but merit further work in other cohorts, including those of individuals with clearly characterized phonological deficits. The limitations of this exploratory study lie in the absence of explicit up-front screening for “atypical development” in addition to the post-hoc identification of individuals with dyslexic traits based on DSM-IV criteria, and the lack of power to detect or grant significance of effects. This work represents a first attempt to look at commonalities and differences in the pattern of correlations with language skills for a range of auditory skills in atypical compared to typical development.

## 5 Conclusion

The present data do not directly support the hypothesis that dyslexia is caused by a simple auditory deficit but suggest subtle differences in the pattern of the ‘yoking’ between auditory and language skills. In view of inconsistent findings from previous studies seeking simple deficits, the approach merits further evaluation. We propose here differences in the pattern of correlations between auditory and language measures that could be tested in further studies. The current data suggest that the relationship between auditory and language skills might differ in subjects within typical vs. atypical language development, with the implication that merging data from differing populations might be problematic. Understanding the relationship between both basic sound feature analysis and music-like sound analysis and language skills in impaired populations might in future suggest appropriate training strategies, possibly including types of musical training to improve language acquisition.

## Figures and Tables

**Table 1:** Descriptive statistics for the twelve auditory measures in the DT group (*n* = 28) compared to the TD group (*n* = 173).

		Typically developing (TD)			Dyslexic traits (DT)			Significance (*p* value)
		Median	MAD	Range	Median	MAD	Range	
Pitch	Basic change (thr. in semitones)	0.85*	0.62	0.07–2.45	−0.60*	0.61	2.43–0.22	n.s.
	Local change (score correct)	29*	4.04	14–39	28.5	3.92	17–37	n.s.
	Global change (score correct)	33*	4.65	16–40	34.0*	4.14	16–38	n.s.
	Key violation (score correct)	21*	3.18	11–30	22.0	2.21	15–28	n.s.
Rhythm	Single-interval duration (thr. in %)	34.0*	20.26	4.0–118.0	33.0*	14.74	14.4–99.0	n.s.
	Isochrony deviation (thr. in %)	15.0*	7.78	3.33–59.0	14.33*	8.16	3.6–56.33	n.s.
	Regularity (thr. in %)	15.75*	4.33	0.5–25.0	17.7	3.6	3.5–24.0	<0.01
	Metrical patterns (thr. in %)	21.0*	8.98	2.0–62.0	18.6*	9.02	2.0–63.0	n.s.
Modulation	2 Hz FM (thr. in MI)	1.68*	0.57	0.62–3.44	1.92*	0.66	0.79–3.44	(0.056)
	40 Hz FM (thr. in MI)	0.074*	0.022	0.028–0.157	0.071*	0.024	0.05–0.154	n.s.
	DM depth (thr. in MD)	0.158*	0.050	0.0–0.696	0.131	0.041	0.058–0.338	<0.01
	DM rate (thr. in cpo)	1.0*	0.694	0.0–3.53	0.78*	0.74	0.15–3.5	n.s.

Pitch: basic change detection using tone pairs; local and global pitch change detection using short sequences; key violation using musical melodies. Rhythm: single-interval duration discrimination; isochrony deviation detection using short sequences; regularity detection using longer sequences; metrical pattern discrimination. Modulation: 2 Hz FM detection; 40 Hz FM detection; DM (dynamic spectral modulation) detection; DM rate discrimination. Shown are the median, mean deviation from the median (MAD), and the range (min to max). Except for three of the pitch tasks that were based on same-different paradigm with fixed difficulty-levels and evaluated in terms of the score correct, all other values correspond to thresholds for detecting an adaptively adjusted difference between the target and the reference. Note that for most of the measure therefore lower values (thresholds) indicate better performance, expect for the three pitch tasks using score correct and the regularity detection task (where the target has an initial value of 0% irregularity that is adaptively changed to approach the reference value of 30%). The thresholds for the rhythm task, were measured as the proportion change in time intervals (which varied in their absolute duration in ms) for the single-interval, isochrony deviation and metrical task, and as the mean jitter value for the target in the regularity task. We report median and MAD, as the majority of measures showed a significant deviation from a normal distribution (Lilliefors Kolmogorov–Smirnoff test; *significant deviation at the level of *p* ≤ 0.05). The significance level for between-group comparisons is given as the uncorrected *p*-value from the Mann–Whitney *U*-Test, given alongside are U and *z* values; none of the comparisons would survive Bonferroni correction for multiple comparison. Abbreviations: thr., threshold; MI, modulation index (proportion change in modulation frequency); MD, modulation depth (0–1, upper limit here, 0.75); cpo, cycles per octave; n.s., non-significant.

**Table 2:** Descriptive statistics for standard measures of phonological and intellectual skills in the DT group compared to the TD group.

	Typically developing			Dyslexic traits			Significance
	Median	MAD	Range	Median	MAD	Range	*p* value
Rhyme decision (PALPA)	55*	4.63	35–66	53	5.78	34–62	<0.05
Spelling (WIAT)	**104**	9.46	73–128	**88**	8.07	68–111	<**0.001**
Word reading (WIAT)	**103**	7.78	75–129	**96**	6.57	77–115	<**0.001**
Non-word reading (WIAT)	103*	8.0	71–121	**95.5**	8.64	65–109	<**0.001**
Non-word repetition (WMTB-C)	97	16.65	57–145	97	14.26	65–145	0.956
Backward digit recall (WMTB-C)	105*	12.36	75–143	98	12.59	68–140	<0.05
Verbal IQ (WASI)	**102**	8.24	77–132	**109.0**	11.46	78–133	<**0.001**
Non-verbal IQ (WASI)	**100.5**	8.47	75–138	**111.5**	7.18	86–126	<**0.001**
Full-scale IQ (WASI)	100.5*	7.09	85–127	**112.5**	8.25	86–133	<**0.01**

All tests were taken from neuropsychological test batteries for children that are named in brackets by their official abbreviations; for a detailed description of tests see main text. Values displayed here are standard scores with a mean of 100 and standard deviation of 15 for all the tests except rhyme decision (max. 66). We report median and MAD, as the majority of measures showed a significant deviation from a normal distribution (*significant at the level of *p* ≤ 0.05; Lilliefors Kolmogorov–Smirnoff test). The significance level for between-group comparisons is given as the uncorrected *p*-value from the Mann–Whitney *U*-Test, given alongside are U and *z* values; comparisons surviving Bonferroni correction for multiple comparison are marked in bold. Abbreviations: PALPA, Psycholinguistics Assessment of Language Processing in Aphasia; WIAT, Wechsler Individual Achievement Test; WMTB-C, Working Memory Test Battery for Children; n.s., non-significant.

**Table 3:** Correlations between auditory and phonological measures in the DT group *before* partialling out non-verbal intelligence.

			Language measures						Mean
			Rym	Spl	Wrd	Nrd	Nrp	Dgb	
Auditory measures	Pitch	Basic pitch change detection	–	–	–	–	0.39	(0.24)	0.23
		Local/global change detection	0.25/0.44	−/−	-/0.33	−/−	-/0.44	(0.27)/-	0.12/0.26
		*Combined* local and global	0.42	(0.23)	(0.28)	–	(0.28)	–	0.26
		Key violation detection	–	(0.24)	(0.28)	–	(0.26)	–	0.18
	Rhythm	Single-interval duration discrimination	–	–	–	–	–	–	–
		Isochrony deviation detection	0.41	–	–	–	–	0.34	0.21
		Regularity detection	–	–	–	–	–	–	–
		Metrical pattern discrimination	–	0.36	(0.25)	–	–	–	0.17
	Modulation	2 Hz FM detection	0.37	–	0.44	–	–	–	0.22
		40-Hz FM detection	–	–	–	–	–	–	–
		DM detection	–	–	–	–	–	–	–
		DM discrimination	0.39	(0.28)	0.35	–	0.35	–	0.24

Listed are the positive Spearman's rho values that explained at least 5% of the variance (rho ≥0.22) and were significant at the level of *p* ≤ 0.05 (none survived Bonferroni correction for multiple comparison); and in addition the mean correlation coefficients across the language measures for auditory measures with at least one individual correlation fulfilling those criteria. In brackets are those with a rho ≥ 0.22, though not significant (but all with *p* values between 0.05 and 0.13), included for comparison with Tables 4 and 5.

**Table 4:** Correlations between auditory and language measures in the DT group *after* partialling out non-verbal intelligence.

			Language measures						Mean
			Rym	Spl	Wrd	Nrd	Nrp	Dgb	
Auditory measures	Pitch	Basic pitch change detection	–	–	–	–	0.39	(0.24)	0.22
		Local/global change detection	–/0.40	−/−	–/(0.24)	−/−	–/0.41	(0.25)/–	–/0.20
		*Combined* local and global	0.38	–	(0.22)	–	(0.24)	–	0.20
		Key violation detection	–	–	–	–	(0.22)	–	–
	Rhythm	Single-interval duration discrimination	–	–	–	–	–	–	–
		Isochrony deviation detection	0.35	–	–	–	–	(0.33)	0.15
		Regularity detection	–	–	(−0.29)	–	–	–	–
		Metrical pattern discrimination	–	(0.31)	–	–	–	–	–
	Modulation	2 Hz FM detection	0.39	–	0.46	–	–	–	0.23*
		40-Hz FM detection	–	–	–	–	–	–	–
		DM detection	–	–	–	–	–	–	–
		DM discrimination	0.35	(0.23)	(0.32)	–	(0.33)	–	0.21*

Listed are the positive Spearman's rho values that explained at least 5% of the variance (rho ≥0.22) and were significant (at the level of *p* ≤ 0.05); and in addition the mean correlation coefficients across the language measures for auditory measures with at least one individual correlation fulfilling those criteria. Listed in brackets are rho ≥0.22, that were not significant (but had *p* values between 0.05 and 0.15), included for comparison with Table 3 and the TD group (Table 5) within which significance is reached easier due to sample size. Asterisks (*) denote those correlations that show a significant deviation (p 0.05, two-sided) from the TD group according to bootstrapping analyses based on 1000 subsamples (abs(*z*) ≥1.96). Abbreviations: Rym, rhyme decision; Spl, spelling; Word, word reading; Nrd, non-word reading; Nrp, non-word repetition; Dgb, backward digit recall.

**Table 5:** Correlations between auditory and language measures in the TD group *after* partialling out non-verbal intelligence.

			Language measures						Mean
			Rym	Spl	Wrd	Nrd	Nrp	Dbg	
Auditory measures	Pitch	Basic pitch change detection	–	–	–	–	–	–	–
		Local/global change detection	−/−	0.22/0.25	−/−	–/0.22	**0.23**/-	−/−	0.17/0.19
		*Combined* local and global	0.22	**0.25**		**0.22**	0.22		0.20
		Key violation detection	–	–	–	–	–	–	–
	Rhythm	Single-interval duration discrimination	–	–	–	–	–	–	–
		Isochrony deviation detection	**0.39**	**0.31**	**0.30**	–	–	–	0.23
		Regularity detection	–	–	–	–	–	–	–
		Metrical pattern discrimination	–	–	–	–	–	–	–
	Modulation	2 Hz FM detection	–	–	–	–	–	–	–
		40-Hz FM detection	–	–	–	–	–	–	–
		DM detection	–	–	–	–	–	–	–
		DM discrimination	–	–	–	–	–	–	–

Listed are the positive Spearman's rho values that explained at least 5% of the variance (rho ≥0.22) and were significant (at the level of *p* ≤ 0.05); and in addition the mean correlation coefficients across the language measures for auditory measures with at least one individual correlation fulfilling those criteria. Marked in bold are those correlations that would survive Bonferroni correction for multiple comparison. Correlations are similar to those reported by Grube et al. (2012), demonstrating that analysing the data from a subsample of 173 (out of 210) in order to match the DT group (by application of a lower limit of IQ, and exclusion of individuals with ASD/ADHD) did essentially not change the results. Abbreviations: Rym, rhyme decision; Spl, spelling; Word, word reading; Nrd, non-word reading; Nrp, non-word repetition; Dgb, backward digit recall.
